# Parliament and the Professions

**Published:** 1921-08-20

**Authors:** 


### Parliament and the Professions.

### Regional Medical Officers.

SIR J. D. REES asked the Minister of Health whether he is aware that the opinion is held by County Councils that the appointment of regional medical officers at £1,000 a year is waste of money, their duties being simply to receive reports of tuberculous cases to pass on to the Council's officers; whether his attention has been called to this matter by the County Councils' Association; and whether he will now review and reconsider, in view of their early abolition, all these appointments. Sir Alfred Mond replied that the duties referred to were but a small part of the main duties of regional medical officers, and he did not think that the Chairman of the Nottingham County Council, who had recently expressed the opinion that as these officers had no duties whatever to perform their salaries were wasted, knows what duties are performed.

### Grants to Voluntary Hospitals.

MR. GILBERT asked the Minister of Health what were the amounts paid in the years 1914 to 1919 respectively as grants-in-aid to the London voluntary hospitals in consideration of their treating sick and wounded soldiers and sailors, and whether it is proposed to publish the evidence given before Lord Cave's Committee. The Financial Secretary to the War Office said that about £880,000 was paid to London hospitals for soldiers' maintenance, and the Minister of Health stated that in view of the cost of printing it was not proposed to publish the evidence given before Lord Cave's Committee.

### Alleged Defects of Health Insurance.

DURING the past ten days the Minister of Health has been asked several questions as to the allegations recently made by the Chief Ranger of the Order of Druids regarding the working of the National Insurance Act, especially as regards certificates being issued by panel practitioners without seeing the insured patients, the tendency to refer patients to hospitals, the reluctance of doctors to call on patients outside surgery hours, and the undesirably large size of panels.

SIR ALFRED MOND answered that he had seen the Press reports of the allegations and was quite prepared to examine specific cases. He is considering whether the time has not come for a general inquiry into the working of the National Health Insurance Acts. Limits of numbers on panels are determined in each district by the Insurance Committee and the Panel Committee jointly. No doctor working single-handed may have more than 3,000 persons on his list. The average number per doctor is approximately 1,000. He would be glad to make inquiries into any specific case in which it is alleged that an Insurance practitioner has a greater number of panel patients than he can adequately attend. He does not see any advantage in making representations to the British Medical Association in regard to the care and attention rendered to Insurance patients by panel doctors. Where it is proved that the continuance of a practitioner on the medical list would be prejudicial to the medical service of the insured, such practitioner is removed; adequate machinery exists and is freely used to deal with offences of a less serious character. Twenty-nine practitioners have been removed from the list since the Act came into operation.

### Registration of Nurses.

SIR J. HOPE asked the Minister of Health whether, under the Rules for Registration of Nurses now lying upon the table of the House of Commons, a nurse who has been trained in Scotland and is practising in Scotland can be admitted to the English Register, and thereby remove herself entirely from the jurisdiction of the Council of the country in which she is practising; and whether he will consider the amendment of both the English and Scottish Rules so that all nurses should originally register in the country in which they are practising, but should be able to be placed on the Register of the other country without difficulty or payment if they subsequently desire to practise in that other country. Sir A. Mond said that he is advised that it is not competent to the General Nursing Council to refuse to admit to the Register a nurse who is otherwise qualified on the ground that she is not practising in the country. Registration is purely voluntary, and no nurse can be brought within the jurisdiction of any of the three Nursing Councils except by her own choice. A nurse practising in Scotland would derive no advantage from admission to the English Register, since she would not thereby be entitled to describe herself in Scotland as a registered nurse.

### Artificial Limbs.

IN reply to Major Cohen and Captain Elliot, the Minister of Pensions (Mr. Macpherson) stated that he understands the report of the Williamson Committee on Artificial Limbs will shortly be submitted to him, and it will be published early thereafter. He is unable to say what conclusions have been arrived at by the Committee, the members of which are Sir Archibald Williamson, Bart (Chairman), Professor A. F. C. Pollard, technical expert; Major Maurice Sinclair, C.M.G., orthopædic and limb-fitting surgeon, Royal Victoria Hospital, Netley; Major M. P. Leahy, disabled (limbless) officer, late R.A.M.C.; Major A. A. Atkinson, disabled (limbless) officer, limb-fitting surgeon at Roehampton; Frank Cecil Meech, disabled (limbless) ex-Corporal of Horse, R.H.G.; Sir Lisle Webb, K.B.E., C.B., C.M.G., D.G.M.S. to the Ministry of Pensions.

### Lady Superintendents at Holloway Prison.

THE Home Secretary (Mr. Shortt) informed Viscountess Astor that of the lady superintendents recently appointed at Holloway Prison one is a highly qualified nurse who has had much experience of outside hospitals. She has the supervision of all the hospital work of the prison and of the hospital staff, subject to the control of the medical officers and the Governor, who is a medical man. The other lady superintendent has had great experience as a prison officer, and has the supervision of the disciplinary side, subject to the control of the Governor.

### Dangerous Drugs Act.

ASKED by Mr. Gilbert whether the regulations under the above 1920 Act have been officially confirmed and are now in operation, and what other Powers have passed legislation in accord with the Opium Convention of 1912 so as to secure co-operation in regard to the import and export of the dangerous drugs named in the Act, Mr. Shortt said the regulations will come into force on September 1. He could give no definite answer as to the second part of the question, but the League of Nations, to which is entrusted the general supervision over the execution of the Opium Convention, is collecting information as to the position in each country.

### Food Supplies at Pensions Hospital.

MR. S. WALSH asked the Minister of Pensions if much dissatisfaction has been caused amongst the patients at the Ministry of Pensions Hospital, Lower Breck Road, Liverpool, in connection with the food supplies served and the manner of cooking and preparation, and whether he would take action to remove the grievance. Mr. Macpherson stated he is having inquiries made.

### Hospitals and Insurance Contributions.

Replying to Sir N. Moore, who asked whether, in view of the work done by hospitals for insured persons, amounts from certain specified funds could be handed over to the Hospitals Commission for distribution among the voluntary hospitals, Sir A. Mond stated that deposit contributors are entitled to the ordinary benefits under the National Insurance Acts only so far as the sum standing to their individual credit will admit, and no question of a valuation arises. No medical funds in respect of deposit contributors remain unallocated. The total amount payable for medical benefits is distributed annually.

**Parliament and the Professions—(*continued*).**

### Lunacy Administration and Treatment.

THE Minister of Health (Sir A. Mond), in reply to Mr. Gould on the subject of articles in the Press in reference to the cure of lunacy by vaccine injections, said there is no obstacle to the use of vaccines in any case in which that form of treatment is considered suitable by the medical authorities of the asylum. Sir Alfred added that he has under consideration the question of possible reforms in lunacy administration and treatment.

### Smallpox, Encephalitis Lethargica and Ophthalmia.

IN reply to Sir B. Chadwick, Sir Alfred Mond stated that there is no reason to attribute the increases in these maladies primarily to the effects of the war, though there is no doubt that the spread and distribution of infectious diseases generally have been influenced thereby. Ophthalmia neonatorum was first made notifiable in April 1914, and encephalitis lethargica in 1919. The notifications of smallpox for 1912, 1913, and 1914 were respectively 121, 113, and 65; and for 1918, 1919, and 1920 were 63, 311, and 280.

### Leavesden Asylum, King's Langley.

SIR R. BLAIR asked the Minister of Health two questions under this head, the first whether, in the impending reduction of the staff at this institution, the principle of juniority will be applied to single men, and whether probationary nurses who have been prevented by sickness from sitting for their examination will be afforded a chance of proving their capabilities before discharge; the second, what steps he proposes to take in the case of Nurse Maclaren, of this asylum, a fully trained and certificated nurse, who, after more than fifteen years' service, has been given notice of dismissal on the ground that her post of superintendent nurse is to be abolished. Sir A. Mond stated that these matters are within the discretion of the managers of the Metropolitan Asylums Board and that the suggestions made are receiving their consideration.

### Bacteriological Examinations.

MR. KENYON asked as to the possible creation of a monopoly in bacteriology in Manchester, as to a request from at least one competent bacteriologist to make arrangements with the local authorities to conduct the same examinations at lower rates than are paid to the main examining body in that city, and whether he would authorise bacteriologists in charge of approved laboratories to enter into arrangements with local authorities for the examination of material. Sir A. Mond replied he had received a request from the bacteriologist referred to, but no application from any local authority to enter into an agreement on the lines suggested. Any such application made on the expiration of existing agreements would receive consideration.

